# Early Gastric Cancer: A Demonstrative Case Report

**DOI:** 10.5005/jp-journals-10018-1099

**Published:** 2014-01-22

**Authors:** Sebahat Basyigit, Bora Aktas, Metin Küçükazman, Hülya Demirkaynak Simsek, Gulçin Güler Simsek, Ayse Kefeli, Abdullah Özgür Yeniova, Yasar Nazligül

**Affiliations:** 1 Department of Gastroenterology, Kecioren Research and Training Hospital, Ankara, Turkey; 2 Department of Gastroenterology, Kecioren Research and Training Hospital, Ankara, Turkey; 3 Department of Gastroenterology, Kecioren Research and Training Hospital, Ankara, Turkey; 4 Department of Pathology, Kecioren Research and Training Hospital, Ankara, Turkey; 5 Department of Pathology, Kecioren Research and Training Hospital, Ankara, Turkey; 6 Department of Gastroenterology, Kecioren Research and Training Hospital, Ankara, Turkey; 7 Department of Gastroenterology, Kecioren Research and Training Hospital, Ankara, Turkey; 8 Department of Gastroenterology, Kecioren Research and Training Hospital, Ankara, Turkey

**Keywords:** Gastroenterology, Endoscopy, Early gastric cancer.

## Abstract

With early detection of gastric cancer, mortality from gastric cancer has decreased. Endoscopists should be more suspicious about these lesions because these can be easily neglected. We reported a case which has endoscopic appearance of benign lesion but possessed malignant histological features.

**How to cite this article:** Basyigit S, Aktas B, Küçükazman M, Simsek HD, Simsek GG, Kefeli A, Yeniova AÖ, Nazligul Y. Early Gastric Cancer: A Demonstrative Case Report. Euroasian J Hepato-Gastroenterol 2014;4(1):61.

## INTRODUCTION

Worldwide, gastric cancer is the second largest cause of cancer-related death. During the past 50 years, incidence and mortality from gastric cancer have decreased worldwide, especially in developed countries.^1^ These improvements can be explained by availability of improved surveillance system and detection of gastric cancer in early stage. Here, we report a case of early gastric cancer that appeared like a benign lesion by endoscopy but found to be of malignant nature by histology.

## CASE REPORT

**Fig. 1:**
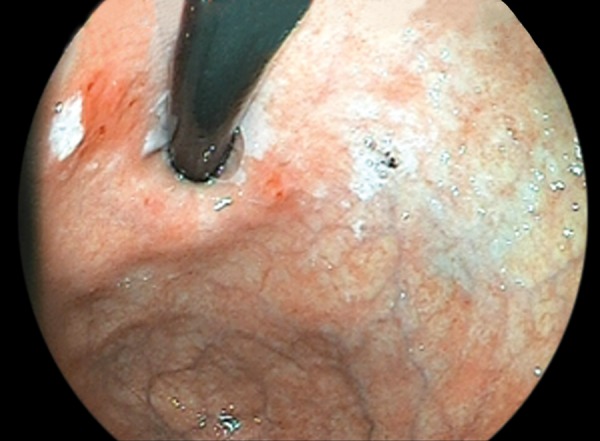
The endoscopic appearance of very small early gastric cancer: a 5 mm in size erythematous area was seen in the cardia

A 71-year-old male patient was admitted to hospital with complaints of fatigue. His physical examination was normal, except pale oral and scleral mucosa. Laboratory examination revealed that hemoglobin level was 10.4 gm/dl and ferritin was 4.9 mg/dl. Biochemical tests were normal. For determining the etiology of the iron deficiency anemia, upper gastrointestinal endoscopy was done. A 5 mm erythematous area was seen in the cardia (Fig. 1). Biopsy was obtained from the lesion. Histopathological examination showed well-differentiated adenocarcinoma from biopsy material.

## DISCUSSION

Gastric cancer is one of the most common forms of malignancy around the world. Its incidence increase with age, and it is seen more common in males.^2^ Early gastric cancers are pathological lesions that are smaller than 2 cm in diameter, irrespective of lymph node metastases. Type 0-I early gastric cancers are polypoid lesions. Type 0-II lesions are nonpoly-poid, type 0-III lesions are excavated.^3^ In our case, early gastric cancer was found to be of type II lesion. This case report indicates that endoscopists must be more suspicious for such lesions that can be easily ignored as malignant lesion.
